# The ecology of *Dunaliella* in high-salt environments

**DOI:** 10.1186/s40709-014-0023-y

**Published:** 2014-12-18

**Authors:** Aharon Oren

**Affiliations:** Department of Plant and Environmental Sciences, The Alexander Silberman Institute of Life Sciences, The Hebrew University of Jerusalem, Edmond J. Safra Campus, Jerusalem, 91904 Israel

**Keywords:** *Dunaliella*, Hypersaline, Halophilic, Great Salt Lake, Dead Sea, Salterns

## Abstract

Halophilic representatives of the genus *Dunaliella*, notably *D. salina* and *D. viridis*, are found worldwide in salt lakes and saltern evaporation and crystallizer ponds at salt concentrations up to NaCl saturation. Thanks to the biotechnological exploitation of *D. salina* for β-carotene production we have a profound knowledge of the physiology and biochemistry of the alga. However, relatively little is known about the ecology of the members of the genus *Dunaliella* in hypersaline environments, in spite of the fact that *Dunaliella* is often the main or even the sole primary producer present, so that the entire ecosystem depends on carbon fixed by this alga. This review paper summarizes our knowledge about the occurrence and the activities of different *Dunaliella* species in natural salt lakes (Great Salt Lake, the Dead Sea and others), in saltern ponds and in other salty habitats where members of the genus have been found.

## Introduction

When the Romanian botanist Emanoil C. Teodoresco (Teodorescu) (1866–1949) described the habitat of the new genus of halophilic unicellular algae *Dunaliella*, it was known from salterns and salt lakes around the Mediterranean and the Black Sea [1-3]. He named the alga in honor of Felix Dunal who in 1838 had described such red-colored unicellular algae growing in the brines of solar salterns [1,3]. Today we known halophilic members of the genus *Dunaliella* to occur in hypersaline environments worldwide as the main and often as the sole primary producer, especially at the highest salt concentrations where other oxygenic phototrophs cannot grow [2,4].

In more than a century since the first description of *Dunaliella*, some representatives of the genus became popular research objects. A search in ISI Web of Science (accessed on 21.9.2014) yielded 1762 papers in which the name features in the title, with ~60 such papers per year in the past decade. This interest is to a large extent due to the exploitation of some halophilic isolates for the commercial production of β-carotene and other valuable products. Some strains are used as model organisms for basic studies in photosynthesis research and salt adaptation. A number of books and review papers have therefore been devoted to the genus [5-7]. However, the ecological aspects of the biology of *Dunaliella* are generally neglected. A recent monograph did not devote a single chapter to ecological aspects, and contained hardly any information about the dynamics and *in situ* activities of natural populations in those ecosystems in which the alga grows [7,8].

I here review the current information about the abundance and the activities of the halophilic members of the genus *Dunaliella* and their interactions with the other components of their hypersaline habitats. Although freshwater and marine species of the genus (which seldom, if ever) are quantitatively important in their ecosystem, they are not discussed here.

## Review

### Taxonomy of the genus *Dunaliella*

In spite of many efforts to establish order, the taxonomy of the genus *Dunaliella* is very confusing [9]. Many of the described and named species were observed only rarely, and the introduction of molecular DNA sequence-based approaches has done little to solve the problems of the classical morphology-based taxonomy.

Older morphology-based studies [10-12] led to the recognition of 28 species, 14 of which can be classified as halophilic with salt optima between 6 and 12%: in the “Section *Dunaliella*”: *D. parva*, *D. salina* and *D. pseudosalina*, and in the “Section *Viridis*”: *D. ruineniana*, *D. gracilis*, *D. bioculata*, *D. carpatica*, *D. granulata*, *D. baasbeckingii*, *D. minuta*, *D. media*, *D. minutissima*, *D. terricola*, and *D. viridis* [12]. This list does not include *D. bardawil*, often considered a form of *D. salina*. This classification was also adopted in the recent monograph on the genus [13]. A re-evaluation of the genus published in 2007 listed 22 species, including 17 halophiles (*D. asymmetrica*, *D. peircei*, and *D. turcomanica*, in addition to the above-listed species). The non-carotenogenic *D. media*, *D. ruineniana*, *D. gracilis*, and *D. baasbeckingii* are known from one field collection only [14].

The species most often reported from natural salt lakes and saltern ponds are *D. salina* and *D. viridis*. Both have a wide salt range, from 9–200 g l^−1^ NaCl to saturation. *Dunaliella viridis* grows optimally at 60–90 g l^−1^ NaCl, *D. salina* at ~100–150 g l^−1^, i.e., concentrations much below the salt concentrations of the environments where halophilic *Dunaliella* species generally thrive [15]. This aspect is further discussed below.

Molecular techniques were introduced in the taxonomy of *Dunaliella* about 15 years ago [13,16,17]. These studies are mostly based on (1) 18S rRNA gene sequences, (2) the number and positions of introns in that gene [17-20], and (3) the sequence of the internal transcribed spacer regions ITS1 and ITS2 in the rRNA gene cluster and the 5.8 S rRNA gene in-between. In addition to sequencing of the genes and gene fragments, fingerprinting techniques such as Restriction Fragment Length Polymorphism were employed [17]. In some studies the gene encoding the large RuBisCo subunit (*rbcL*) was also included [21].

Sequence comparisons confirmed the monophyly of the subgenus *Dunaliella* [22], but within this subgenus *D. viridis* was found to consist of at least four clades and *D. salina* of three [23]. It became clear that many culture collection strains had been mislabeled [17]. DNA fingerprinting and intron-sizing methods may be suitable for specific, rapid, and sensitive identification of *Dunaliella* species [19,24], but no correlation was shown between the genetic relationship inferred from ITS-RFLP data and the morphological and physiological attributes [16].

In ecological studies, such molecular methods were seldom exploited to obtain information about the population structure of *Dunaliella*. The technique was used in a biogeographic study of *D. salina*: sequence comparison of the primary and secondary structure of the ITS2 sequences and Compensatory Base Changes analyses (where both nucleotides of a paired site mutate while pairing is maintained) did not yield clear geography-related signals [25].

Culture-independent small-subunit rRNA-based and metagenomic studies were performed in many hypersaline environments, including Great Salt Lake, the Dead Sea, and solar salterns. However, such studies did not contribute to our understanding of the structure of *Dunaliella* communities in such ecosystems. Most environmental genomic studies targeted “prokaryotic” 16S rRNA genes, neglecting eukaryotic 18S rRNA genes. The study of 18S rRNA sequences along two depth profiles in Great Salt Lake in 2007 [26] is a rare exception. As numbers of *Dunaliella* and other eukaryotes are typically four or more orders of magnitude lower than those of the prokaryotes in the community, *Dunaliella*-derived genes are never conspicuously present in metagenomic libraries. For example, no *Dunaliella*-related sequences were found in fosmid clones prepared from DNA extracted from the 1992 microbial bloom in the Dead Sea [27]. A notable exception is the characterization of eukaryotic microbial diversity in hypersaline Lake Tyrrell, Victoria, Australia, based on 18S rRNA gene inventories [28], further discussed below.

The earliest descriptions of *Dunaliella* mentioned the possibility of formation of palmelloid cells and resting cysts (aplanospores). Encysted zygotes resulting from gamete union in sexual reproduction were also described [10,29]. Under non-optimal salt concentrations caused by drastic dilution of the medium or drying-up of the environment, cells may form asexual thick-walled non-motile cysts with bumpy surfaces, often referred to as aplanospores [9,12,14,30]. In *D. salina*, such aplanospores contain canthaxanthin as the main carotenoid rather than β-carotene found in the vegetative cells [15]. In a study of *D. salina* from saline environments of the central coast of Peru, formation of spherical, reddish aplanospores were observed when collected plankton samples were kept in the laboratory for a few days [31]. In Great Salt Lake, round cyst-like cells of *D. salina* increased in numbers, especially on the bottom, when the temperature decreased below 0°C [32]. In the Dead Sea such cysts may play a key function in long-term survival of *Dunaliella* in a lake that is now generally too extreme to support algal growth (see below). Remote sensing showed that the bloom that started in April 1992 following dilution of the upper water layers originated in the shallow near-shore areas. It was probably derived from resting cells that survived in the shallow sediments [33]. Formation of thick-walled cysts was observed during the decline of this bloom [34].

Palmelloid stages, in which non-motile cells are encased in a gelatinous mass of mucus, may also form under certain conditions [10,12]. In hypersaline pools of 16.5–35% salt on the central coast of Peru, benthic palmelloid stages of *D. salina* were found at the higher salinities. Palmelloid benthic forms were seen mainly in winter at depths up to 1 m as irregular and lobulated clusters up to 3 mm in diameter. Nutrient deficiency, low temperature and low salinity may trigger the production of these palmelloid forms [31].

### *Dunaliella* in hypersaline environments – Case studies

#### Great Salt Lake

Surprisingly, little recent information is available about *Dunaliella* in Great Salt Lake, where it is the main (or the only) primary producer in the north arm and an important component of the phototrophic community in the south arm. Both green *D. viridis* and β-carotene-rich *D. salina* play a role in the ecosystem. Most studies date from the 1970s and 1980s, and these were reviewed earlier [32,35]. Two papers published in 2013 [26,36] do little to complete the picture. This is to be regretted as Great Salt Lake is a dynamic ecosystem that has undergone dramatic changes in water level and accordingly in salinity in the past decades. The lake is divided by a railway causeway into a less saline southern part and a hypersaline north arm that now approaches NaCl saturation.

In the 1970s, *D. salina* was the dominant planktonic alga in the north arm (~332 g l^−1^ salt), with typical population densities of 200–1000 cells ml^−1^ and peak densities of 3000 to 10000 cells ml^−1^. Its horizontal distribution was highly patchy. *Dunaliella viridis* was found in the north arm mostly on the underside of rocks and wood along the shallow margin, out of direct sunlight [32,37]. In the water column, *D. viridis* densities were sometimes higher in the deeper layers than at the surface. Thus, a density of 4000 cells ml^−1^ was recorded at 4.5 m depth in the north arm in August 1975, an order of magnitude higher than the numbers in the upper 1.5 m. Whether this vertical distribution was caused by supraoptimal light intensities at the surface or by nutrient availability is unknown. Ammonium is the key nutrient controlling biological processes in the lake [32,38]. When (in the summer of 1977) a severe drought caused the north arm to shrink and a crust of NaCl formed on the shore, massive development of *Dunaliella* below the 3–4 cm thick salt crust was observed with up to 35000 *D. salina* and 2000 *D. viridis* cells g^−1^ salt. Oxygen production by the algae led to the elevation of parts of this crust as 7–15 cm-large ‘salt domes’ [39].

In the less saline (~117 g l^−1^) south arm, *D. viridis* dominated the phytoplankton in the 1970s, with densities exceeding 1000 cells ml^−1^. Short-lived blooms with up to 25000 cells ml^−1^ were recorded in the spring of 1971 and 1973 [32,40].

Primary production measurements based on ^14^CO_2_ incorporation in the water column at two stations in the south arm in 1973 yielded an estimate of ~145 g C m^−2^ year^−1^. The highest activity was recorded during the *Dunaliella* bloom in March-April (2.13 g C m^−2^ day^−1^). In winter, temperatures were too low; in summer, production was limited by nitrogen availability and by grazing by the brine shrimp *Artemia* [40].

The 1970s and 1980s were a period of increased precipitation in the area. As a result the salinity of Great Salt Lake decreased greatly. By 1986, the salt concentration in the south arm had dropped to ~60 g l^−1^. Halophilic *Dunaliella* species were outcompeted by other phototrophs, especially the cyanobacterium *Nodularia* [41]. Then the lake level dropped again, and in the last decade the salt concentrations in the south and the north arm were around 140–150 g l^−1^ and 270–300 g l^−1^, respectively.

In recent years, in-depth molecular studies of the biota of Great Salt Lake were initiated, aimed at an understanding of the prokaryote diversity. Eukaryotic diversity, as monitored by 18S rRNA gene sequences recovered from environmental DNA, was included in an analysis of a depth profile at one station in the south arm and one station in the northern part in June 2007. *Dunaliella*-related sequences formed 71-96% of the eukaryote sequences in the south arm profile (0, 4, 6, 6.5 and 8 m depth) [26]. Microcosms (400 ml) were set up with water from the south arm to study the effect of salinity and nutrient levels on the biota. The relative abundance of *D. viridis* increased with increasing salinity (up to 150 g l^−1^), especially at the lower temperatures tested (10–20°C) [36].

### The Dead Sea

Currently, no *Dunaliella* cells can be found in the waters of the Dead Sea. The negative water balance and the increase in divalent cation concentrations (>2 M Mg^2+^, ~0.5 M Ca^2+^) has made the lake an environment too hostile even for the most salt-tolerant algal genus. But in the past, *Dunaliella* has been present in large numbers during periods when the salinity of the upper water layers was lower than today. Its development has at least twice triggered the development of dense blooms of red halophilic Archaea: in 1980 and in 1992, after dilution of the upper meters of the water column following unusually rainy winters [42,43]. *Dunaliella* cells, identified as *D. viridis*, were first cultured from Dead Sea water in enrichment cultures with 75% Dead Sea water and 25% fresh water in the late 1930s [44,45]. Later isolates were identified as *D. parva* [46,47]; see [42] for further comments about the identification at the species level. Red, β-carotene-rich types were never encountered. Development of *Dunaliella* in the Dead Sea is restricted not only by the generally too high salinities and the unfavorable ionic composition of the brine, but also by lack of phosphate, the limiting nutrient [48].

Quantitative information about the population dynamics of *Dunaliella* in the Dead Sea prior to 1980 is scarce. Up to 40000 cells ml^−1^ were reported in surface water in 1964 (sampling date not specified), when the lake was still meromictic (a period that ended with the overturn of the water column in February 1979). At 50 m depth numbers were two orders of magnitude lower, and no algal cells were encountered at 100 m depth [49]. A bloom of up to 8800 cells ml^−1^ developed in the upper 5–10 m of the water column in the summer of 1980 after massive amounts of rain water had entered the lake during the preceding winter, starting a new meromictic episode that lasted until the end of 1982. The algal population was distributed evenly over the mixed layer above the pycnocline [47]. Then, a period of ten years followed, in which no *Dunaliella* cells were observed in the many water samples examined. The upper meters of the lake’s water column once more became greatly diluted, this time down to ~70% of the original salinity, during the rainy winter of 1991–1992. A new *Dunaliella* bloom rapidly developed in April-May 1992 with densities up to 15000 cells ml^−1^, evenly distributed down to the pycnocline located at 5 m depth [34]. As explained above, thick-walled cysts found within the shallow sediments may have formed the inoculum that enabled the development of the new bloom; the lowered salinity probably triggered their germination [33]. From August 1992 onwards, *Dunaliella* cells started to appear near the pycnocline in numbers up to 1850 cells ml^−1^; a year later this ‘deep chlorophyll maximum’ was found at 14 m depth (~3000 cells ml^−1^). Occurrence of healthy, motile *Dunaliella* cells at these depths, at low light intensities and at salinities generally considered too high for growth, may be related to the availability of nutrients, especially phosphate that may have been depleted in the surface waters [34]. During the 1992 bloom, attempts were made to estimate primary production using ^14^C-bicarbonate as a tracer. Maximum estimated values recorded in May, at the peak of the bloom, were ~142 mg C l^−1^ day^−1^ and an integrated value of ~0.66 g C m^−2^ day^−1^. These measurements were supplemented with stable isotope studies in which changes in the ^13^C content of the dissolved inorganic carbon were related to biological phenomena [34].

After a new overturn of the water column in the end of 1995, ending a four-year meromictic episode, conditions in the Dead Sea never became suitable again for development of *Dunaliella*.

### Other natural lakes

The community of *Dunaliella* and other eukaryotes in Lake Tyrrell, an ephemeral thalassohaline lake near Melbourne, Australia, was characterized using molecular techniques. In the water column *Dunaliella* represented 3.4% of the sequences in summer (250–300 g l^−1^ salt) and 2.4% in winter (>330 g l^−1^ salt). The dominant planktonic eukaryote was a novel type of the Alveolate *Colpodella*. But within the up to 7-cm thick halite crust that formed in winter, 91.1% of the 18S genes detected in clone libraries were related to *D. salina*, *D. parva*, and *D. viridis*. The halite crust may provide a refuge from predation, leading to dominance of *Dunaliella* [28].

A survey of 53 saline lakes in Antarctica showed *Dunaliella* to be present in three lakes only: Deep Lake and Lake Stinear (Vestfold Hills) and Lake Hunazoko in Skarvs Nes [50].

### Solar salterns

*Dunaliella* was first spotted in saltern evaporation and crystallizer brines [1-3,29] and relatively much information is available about the occurrence of the genus in salterns worldwide [51-55]. In crystallizer ponds, *D. salina* is found in numbers that vary from ~150 to ~3000 cells ml^−1^ in the oligotrophic salterns of Eilat, Israel up to a maximum of 100000 cells ml^−1^ recorded in a Spanish saltern pond [55]. In extremely oligotrophic salterns, *D. salina* may be altogether absent [56,57]. Growth can be highly patchy: in a single pond of the Santa Pola salterns (Alicante, Spain) with 370 g l^−1^ salt, cell numbers varied between 5090 and 10500 per ml, with chlorophyll *a* concentrations between 23.6 and 45.7 μg l^−1^ [54]. A study in evaporation ponds of relatively low salinity (up to 144 ppt) at the Megalon Embolon saltworks in northern Greece showed *D. salina* to represent 5–22% of the total microalgal assemblage in spring; values decreased to 0.3–1% during summer as a result of grazing by the brine shrimp and ciliate protozoa. Salinity and phosphate availability were further identified as the main factors affecting *Dunaliella* growth [58].

*Dunaliella salina* with its high β-carotene content is not the only red-pigmented microorganism in saltern crystallizer brines: it is accompanied by a community of halophilic Archaea (family *Halobacteriaceae*), colored pink-red by the carotenoid α-bacterioruberin and derivatives and possibly also by bacteriorhodopsin and other retinal-containing membrane proteins. An additional pigment that may be present is salinixanthin of *Salinibacter* (*Bacteroidetes*) [59]. In all systems investigated the pigment present in the largest amounts in such brines is β-carotene. It is densely packaged as globules within the interthylakoid space of the cell’s single chloroplast. As a result it contributes relatively little to the overall color of the brine, which is dominated by bacterioruberin of the Archaea. For quantitative recovery of the pigments, filtration is the appropriate method; during centrifugation of the brine, *D. salina* cells often tend to float, so that very little β-carotene is recovered in the pellet [60-62].

Polysaccharides excreted by the biota of saltern ponds can negatively affect the quality and the quantity of the salt produced. Most problems caused by accumulation of polysaccharides in crystallizer brines are due to massive development of unicellular cyanobacteria (*Aphanothece*) at lower salinities. However, *D. salina* may also produce extracellular polymeric substances, especially at elevated salt concentrations [63]. There are reports showing that release of organic substances by *Dunaliella* may negatively affect size and quality of the NaCl crystals formed [64-66]. However, to what extent accumulations of *Dunaliella* rather than of cyanobacteria are the true cause of the production of poor quality salt remains unclear.

### Hypersaline soils and spider webs in desert caves

*Dunaliella* is a typically aquatic genus. However, a community of *Dunaliella* was found in hypersaline soils of the Great Salt Plains, Oklahoma. It included motile single cells, a palmelloid phase with non-motile cells and mucilage, and palmelloid forms with weak motility. Based on 18S rRNA sequence data the diverse lineages found were affiliated with *D. viridis* [67].

Even more unexpected is the finding of a novel subaerial *Dunaliella* species growing on spiderwebs on the wall of a cave in the Atacama Desert, Chile. The entrance to the cave confronts the Pacific Ocean, receiving more humid air coming from the ocean. The algae exploit the air moisture condensing on the spiderweb silk threads. Based on gene sequence information (18S rRNA, chloroplast 16S rRNA, the photosystem I reaction center gene *psaB* and the large RuBisCo subunit *rbcL*), the organism is a member of the *Dunaliella* genus, and it was named *D. atacamensis*. Thus far it has not been brought into culture, and its salt requirement and tolerance are yet unknown [68].

### Brine inclusions in crystals of halite or gypsum

When salt crystallizes from NaCl-saturated solutions, the halite crystals often contain brine inclusions. Microorganisms living in the brine can become trapped in these inclusions. Different types of halophilic prokaryotes, Archaea as well as Bacteria, could be revived from brine inclusions within salt deposited tens to hundreds of thousands of years ago. *Dunaliella*-like cells are may also be found within such brine inclusions. Thus, remains of large carotenoid-rich cells resembling *D. salina* were observed within 10–34 k.y. and 100 k.y. old halite crystals in a core from Death Valley, CA. Presence of algal cells within brine inclusions explains in part the longevity of the prokaryotes present: the dying algae may have supplied an abundant source of carbon and energy, including a large amount of glycerol accumulated for osmotic stabilization [69-73].

Pale-yellow pigmented algal cells were observed within fluid inclusions entrapped in halite deposited from Lake Magic, an ephemeral acidic (pH 1.7) and hypersaline (~325 g l^−1^ salt) lake in Western Australia [74]. These cells were interpreted as *Dunaliella* algae. Such algae were also found, together with pennate diatoms and different types of prokaryotes, within gypsum crystals precipitated from acid (pH 1.8–4.6) saline (5–28%) water at Salars Gorbea and Ignorado in the high Andes of northern Chile [75]. Also here, further study of the taxonomic affiliation of these algae is necessary.

### *In situ* activities of *Dunaliella* in hypersaline environments

The above survey of hypersaline habitats where *Dunaliella* spp. are found demonstrates that the alga is generally found at salinities much higher than the optimum values for growth. In culture, *D. salina* grows optimally at ~100–150 g l^−1^ salt, and *D. viridis* at ~60–90 g l^−1^. In nature they are seldom found at these salinities as they are outcompeted by other, faster-growing phototrophs, eukaryotic as well as prokaryotic. Thanks to their ability to grow at salt concentrations up to saturation, *Dunaliella* species dominate the ecosystem at the highest salinities where they lack competitors [15,66,76].

While in the athalassohaline salt-saturated environment of the Dead Sea only smaller green types were found, the large-celled *D. salina* generally outcompetes *D. viridis* and *D. parva* in NaCl-dominated brines approaching saturation. This is illustrated in a study in Hutt Lagoon, a hypersaline lagoon in Western Australia where *D. salina* is grown for β-carotene production. In summer, both species survive below the halite layer of the dry lake bed. Upon winter flooding the cells are released into the water column. When the salt concentration increases above 250 g l^−1^, *D. viridis* migrates to the bottom, but *D. salina* remains active in the water column up to a salt concentration of 310 g l^−1^. Thanks to its high carotenoid content, *D. salina* is better adapted to high light intensities than *D. viridis* [77].

It is surprising how little we know about the activity of *Dunaliella* populations in their natural habitats. There are only few estimates of primary production and *in situ* growth rates. This is to a large extent due to methodological constraints [51]. Cells are fragile, and during filtration the cells are ruptured on the filter, releasing their contents. β-carotene-rich *D. salina* cells cannot be collected by centrifugation [60]. For ^14^C-tracer experiments, essential information about the carbonate system of the brines and the availability of different forms of inorganic carbon for photosynthesis is generally lacking, and conventional oxygen electrodes perform poorly - if at all - in saturated brines. Therefore, it is difficult to assess the reliability of the few estimates of annual production of hypersaline lakes found in the literature (e.g. ~25 g C m^−2^ year^−1^ for Pink Lake, Victoria, Australia, ~200 g C m^−2^ year^−1^ for Great Salt Lake [78], and <10 g C m^−2^ year^−1^ for Deep Lake, Antarctica [79]).

In a study of the saltern ponds near Alicante, Spain, photosynthetic activity was monitored along the salinity gradient using ^14^C-bicarbonate as a tracer and 3 hrs incubation periods. Maximum carbon incorporation rates were obtained in the low salinity (80 g l^−1^) ponds. However, in the crystallizer ponds populated by a dense *Dunaliella* population with a high chlorophyll content very low uptake rates were measured. In brine of 370 g l^−1^ salt, rates of 27.5-56 μg C l^−1^ day^−1^ were recorded in the light and 20.8-23.8 μg C l^−1^ day^−1^ in the dark, values corresponding to ~0.1 μg C per μg chlorophyll *a* h^−1^. These low values were attributed to the heavy stress due to the supraoptimal salinity [54]. But the low values reported may also, in part, have been an artifact due to cell lysis during vacuum filtration on 0.2 μm polycarbonate filters employed in the study [51], leading to an underestimation of the true carbon fixation rates. In another study of the same saltern, primary production in the crystallizer ponds was below detection limit, despite the presence of 3.5 μg l^−1^ chlorophyll *a* of *Dunaliella*. In ponds of intermediate (200–250 g l^−1^) salinity with ~3.6 μg l^−1^ chlorophyll *a* and 74–96% of the biovolume being *Dunaliella* (cyanobacteria being present as well at 4–9%), gross production was estimated at 0.07–1.27 μg C per μg chlorophyll *a* day^−1^ [80].

There also have been attempts to estimate the *in situ* activity of *Dunaliella* in saltern brine mesocosms by measuring changes in oxygen concentrations, using a chemical assay (a modification of the Winkler titration) or by means of electrodes. Monitoring diel changes in dissolved oxygen concentrations in mesocosms with crystallizer brine from the salterns of Eilat, Israel (1300–2100 *D. salina* cells ml^−1^) yielded production estimates of ~0.8–1.5 μmol O_2_ l^−1^ hr^−1^ [51,81]. These rates, equivalent to ~120–220 μg C l^−1^ day^−1^, are significantly higher than those measured in the Spanish salterns populated by denser *Dunaliella* communities [54]. In another mesocosm experiment with Eilat crystallizer brine (1100 *D. salina* cells ml^−1^) incubated at 35°C in the light and in the dark, changes in oxygen concentration were monitored using an optical oxygen sensor (optode). Calculated production rates were ~1 μmol O_2_ l^−1^ hr^−1^, equivalent to ~9 × 10^−13^ mol O_2_ cell^−1^ hr^−1^ (R. Pinhassi, E. Maimon, and A. Oren, unpublished results).

*Dunaliella* is the key toward the understanding of the functioning of most hypersaline ecosystems as it is the main or sole primary producer on which the heterotrophic components of the ecosystem depend. One of the key compounds linking the activity of the alga with that of the consumers is glycerol, accumulated by *Dunaliella* in molar concentrations intracellularly as an osmotic stabilizer [15,79,81]. To what extent glycerol may diffuse through the membrane of healthy *Dunaliella* cells is not completely clear. Some studies showed a very low permeability of the *Dunaliella* membrane to glycerol, but others claim that also in healthy cells significant amounts of glycerol may diffuse continuously into the medium [82]. At temperatures above 40°C, as regularly encountered in saltern evaporation ponds [55], glycerol leakage maybe significant [83].

## Conclusions

In spite of the in-depth understanding of the properties of *Dunaliella* strains used for the commercial production of β-carotene, often under carefully controlled conditions, we know relatively little about the behavior of these species and their closest relatives in their natural environment or in semi-natural systems such as the saltern ecosystem. This is at least in part due to the lack of appropriate methods to study these fragile organisms. But also the potential established with the development of molecular, gene, genomics and proteomics based methods was rarely exploited thus far in the study of the ecology of *Dunaliella*. The (relative) simplicity of hypersaline ecosystems presents a huge advantage for ecological studies, and *Dunaliella* is a key component of these ecosystems. Therefore, further exploration of the functioning of *Dunaliella* in salt lakes and saltern ponds may add much important information, not only toward the understanding of the hypersaline environments, but also for microbial ecology in general.
